# Impact of 3–Aminopropyltriethoxysilane-Coated Iron Oxide Nanoparticles on Menaquinone-7 Production Using *B. subtilis*

**DOI:** 10.3390/nano7110350

**Published:** 2017-10-26

**Authors:** Dinali Ranmadugala, Alireza Ebrahiminezhad, Merilyn Manley-Harris, Younes Ghasemi, Aydin Berenjian

**Affiliations:** 1 Faculty of Science and Engineering, University of Waikato, Hamilton 3216, New Zealand; dinalir@yahoo.com (D.R.); merilyn.manley-harris@waikato.ac.nz (M.M.-H.); 2 Department of Medical Biotechnology, School of Medicine and Noncommunicable Diseases Research Centre, Fasa University of Medical Sciences, Fasa 74615, Iran; 3 Department of Pharmaceutical Biotechnology, School of Pharmacy and Pharmaceutical Sciences Research Center, Shiraz University of Medical Sciences, Shiraz 71348, Iran; ghasemiy@sums.ac.ir

**Keywords:** MK–7, *B. subtilis*, IONs@APTES, immobilization, fermentation

## Abstract

One of the major issues associated with industrial production of menaquinone-7 (MK–7) is the low fermentation yield. In this study, we investigated the effect of iron oxide nanoparticles coated with 3–aminopropyltriethoxysilane (IONs@APTES) on the production of MK–7 using *B. subtilis* (ATCC 6633). Decoration of *B. subtilis* cells with IONs@APTES significantly enhanced both MK–7 production and yield. An approximately two-fold increase in MK–7 production (41 mg/L) was observed in the presence of 500 µg/mL IONs@APTES, as compared to MK–7 production using untreated bacteria (22 mg/L). This paper, therefore, illustrates the immense biotechnological potential of IONs@APTES in increasing MK–7 concentration using *B. subtilis*, and its future role in bioprocess engineering.

## 1. Introduction

In terms of human health, the intake of MK–7 plays a vital role in bone formation [1], reducing bone fractures [2], and in preventing postmenopausal bone loss by improving bone mineral calcification and femoral neck width [3]. In addition, a direct link has been observed between MK–7 consumption and improved cardiovascular health. In particular, supplementation of MK–7 has been shown to decrease arterial stiffness in postmenopausal women [4]. Therefore, interest in MK–7 has increased considerably over the past years, and it can be regarded as an obvious choice to be included in multivitamin supplements for the prevention of these diseases [2]. 

MK–7 can only be produced thorough a fermentation process. Among the bacterial species used in MK–7 production, *B. subtilis* is one of the most studied and recognized MK–7 producers [5]. Although many studies on MK–7 production have been published using *B. subtilis*, the fermentation process is still not sustainable enough. One of the complications is low fermentation yield [6]. 

Currently, nanoparticles (NPs) are at the forefront in designing intensified bioprocesses with the combined advantages of reducing downstream processing and maximizing the production of the target products [7]. In terms of bioprocess intensification, NPs have been used to decorate bacterial cells and magnetically separate and re-use them in the fermentation system, which essentially eliminates downstream purification steps such as filtration and centrifugation [7,8]. Stepping towards the designing of an intensified MK–7 production system, recently, *B. subtilis* cells were magnetically immobilized with naked iron oxide nanoparticles (IONs) and subsequently recovered from the fermentation media with a high capture efficiency [9]. In addition to reducing the number of downstream processing steps, magnetic immobilization of *B. subtilis* offers the advantages of good mass transport, catalytic stability [10] and improved metabolic activity [7]. Nevertheless, immobilization with naked IONs resulted in lower cell densities and metabolic activity [9]. Further, when naked IONs are used in biological systems, many problems are encountered, such as particle agglomeration, low stability, altered magnetic properties [11], and cytotoxicity [12]. As the interaction of bacteria with nanoparticles is governed by the properties of the bacterial cell wall, as well as the physico-chemical characteristics of the nanoparticles, it is crucial to choose IONs with surface properties that importantly fulfill the fermentation goals; namely, high metabolic activity and biocompatibility.

Recent studies have shown that amine-functionalized nanoparticles have more biological benefits than naked particles, due to their biocompatibility, surface activity, stability and chemical simplicity [12,13,14]. Compounds such as l-lysine, l-arginine and 3–aminopropyltriethoxysilane (APTES), which introduce amine functional groups to the nanoparticles, have been used to coat negatively charged nanoparticles, thereby increasing the chance of binding nanoparticles to the anionic cell membrane. Previously, IONs coated with l-lysine had been reported to result in no significant inhibitory effect on MK–7 production and cell growth [7]. However, it has now been reported that, in comparison with l-lysine and l-arginine, coating of nanoparticles with APTES prevents the oxidation of nanoparticles, and provides perfect protection for the crystal structure of nanoparticles [13]. Therefore, the present study aimed to synthesize iron oxide nanoparticles coated with 3–aminopropyltriethoxysilane (APTES) and evaluate their effect on MK–7 biosynthesis during *B. subtilis* fermentation.

## 2. Results and Discussion

### 2.1. Synthesis and Characterization of Iron Oxide Nanoparticles Coated with APTES

**Figure 1:**
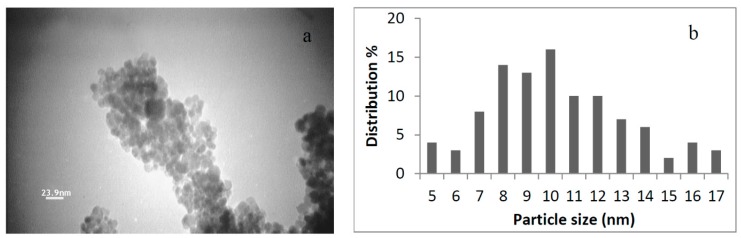
(**a**) Transmission electron micrograph of IONs@APTES; and (**b**) Particle size distribution of IONs@APTES.

The Transmission Electron micrograph of IONs@APTES is presented in Figure 1, showing the spherical-shaped IONs@APTES with a size distribution ranging from of 5 to 17 nm.

**Figure 2:**
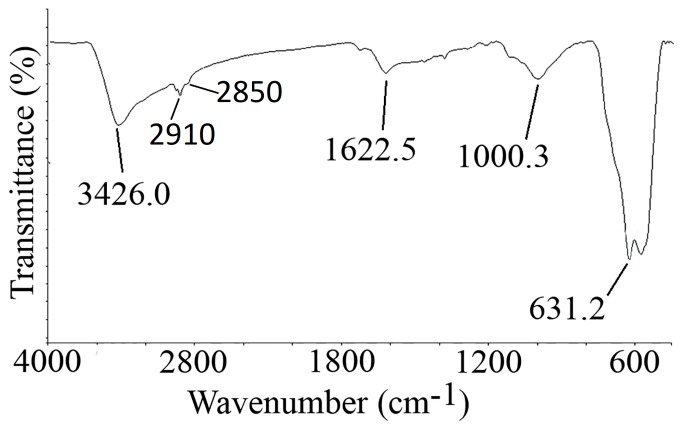
Fourier transform infrared spectroscopy (FTIR) spectra of IONS@APTES.

Figure 2 represents the Fourier transform infrared spectroscopy (FTIR) spectra of IONs@APTES. The Fe–O characteristic peaks of IONs@APTES appeared at about 631.2 cm^−1^. The Si–O bond stretching vibration appeared at 1000.3 cm^−1^ in IONs@APTES [15]. Peaks at 1622.5 cm^−1^ and 3426 cm^−1^ are attributed to O–H bending and stretching vibrations of OH groups [15,16,17,18,19]. Coating of IONs with APTES is established by the presence of peaks at 2910 and 2850 cm^−1^, which can be attributed to the asymmetric and symmetric –C–H stretching vibrations, and are indicative of the presence of aliphatic –CH_2_ groups. 

**Figure 3:**
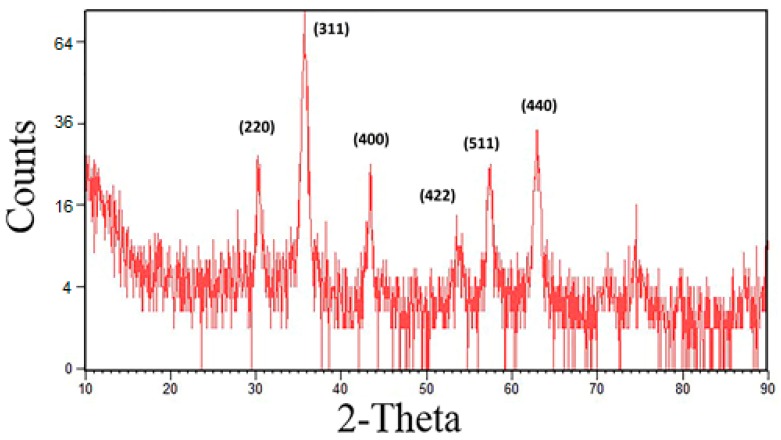
XRD patterns of IONs@APTES.

X-Ray diffraction analysis shows major intensity peaks at 2θ degrees 30°, 35.5°, 43°, 53°, 57° and 62°. In comparison to standard data for magnetite crystals, the peaks correspond to 220, 311, 400, 422, 511 and 440 Bragg reflections, confirming the spinal structure of magnetite (Fe_3_O_4_). The 2θ value for the peak at 35.5° confirmed the predominant magnetite (Figure 3). The crystal size was calculated by using the Scherrer calculator toll on the PANalytical X’Pert HighScore (Produced by PAN alytical B.V., Almelo, The Netherlands, version 1.0d), showing a size of 14 nm.

### 2.2. Interaction of B. subtilis Cells with IONs@APTES

**Figure 4:**
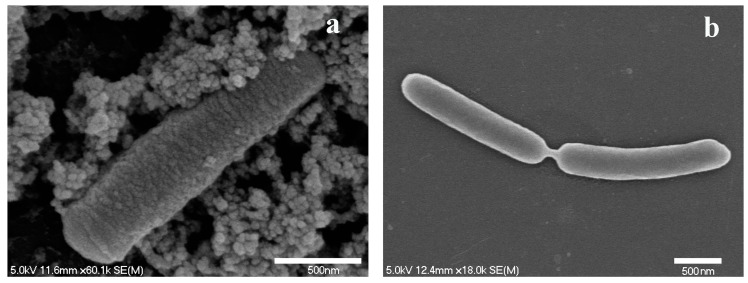
(**a**) Interaction of IONs@APTES with *B. subtilis*; (**b**) *B. subtilis* in the absence of IONs@APTES.

Scanning Electron Microscopy (SEM) was used to observe the interaction of IONs@APTES with *B. subtilis* cells. IONs@APTES, with their smaller size, high surface area/volume ratio and positively charged amine groups, were readily attached to *B. subtilis* cells when they were grown in the presence of varying IONs@APTES concentrations. Figure 4 illustrates the successful decoration of *B. subtilis* with IONs@APTES in comparison with untreated cells.

### 2.3. Effect if IONs@APTES on MK–7 Production

**Figure 5:**
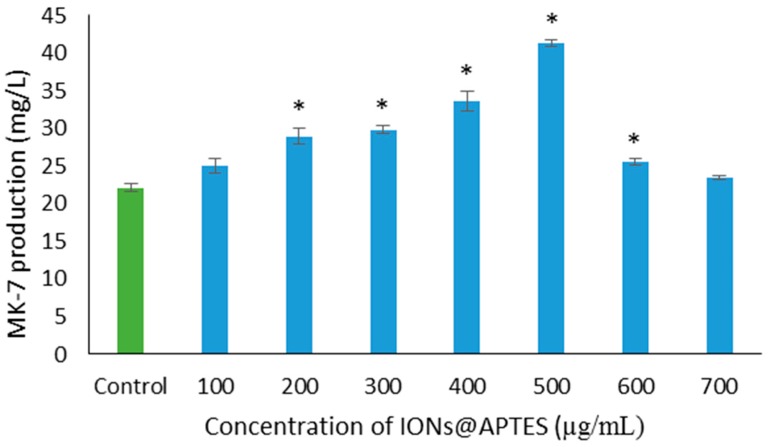
MK–7 production by *B. subtilis* in the presence of varying concentrations of IONs@APTES and in the absence of nanoparticles. Values are mean ± S.E. of three replicates. An *asterisk* indicates a significantly different value from the control (*p* < 0.05).

To explore the effect of IONs@APTES on menaquinone production by *B. subtilis*, MK–7 production was compared in the absence and the presence of varying concentrations of IONs@APTES (100–700 µg/mL). The relationship between IONs@APTES attachment to *B. subtilis* cells and their effect on MK–7 production is shown in Figure 5. When the concentration of nanoparticles was increased from 100 µg/mL to 500 µg/mL in the fermentation media, MK–7 production increased from 22 to 41 mg/L. However, when the concentration of IONs@APTES was increased beyond 600 µg/mL, the increase in MK–7 production was not significantly different from the control sample. The highest MK–7 production of 41 mg/L was 2-fold higher as compared to untreated bacteria. These results show the key role played by decorating *B. subtilis* cells with IONs@APTES in enhancing the MK–7 biosynthesis. 

### 2.4. Effect of IONs@APTES on B. subtilis Growth

**Figure 6:**
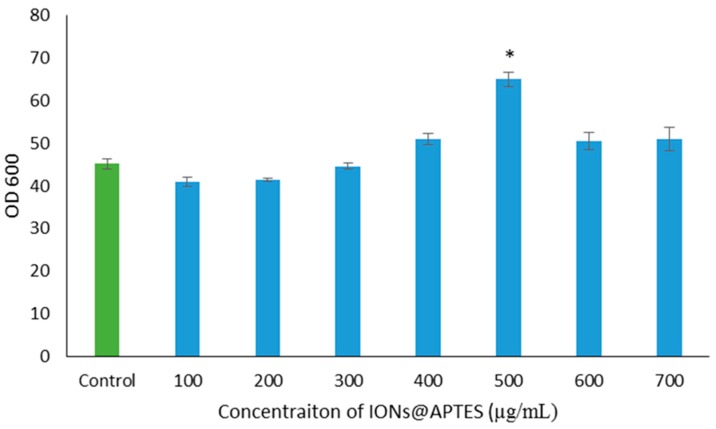
Growth of *B. subtilis* in the presence of IONS@APTES and in the absence of nanoparticles. Values are mean ± S.E. of three replicates. An *asterisk* indicates a significantly different value from the control (*p* < 0.05).

The effect of IONs@APTES on *B. subtilis* growth was also investigated. In this regard, cells were grown for 60 h in the presence of seven different concentrations of IONs@APTES (100–700 µg/mL) and in the absence of nanoparticles (Figure 6). Based on the results, presence of IONs@APTES shows different effects on *B. subtilis* growth. An increasing trend in *B. subtilis* cell densities was observed with increase in IONs@APTES concentration up to 500 µg/mL. However, there was no statistically significant difference between the samples decorated with 100–400 µg/mL as compared to the control samples. Concentrations higher than 500 µg/mL of IONs@APTES also led to a decrease in *B. subtilis* cell density.

### 2.5. Effect of IONs@APTES on MK–7 Yield

**Figure 7:**
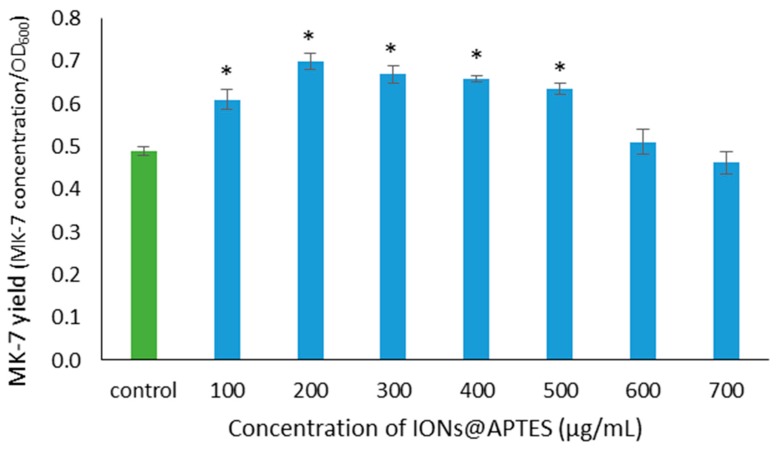
MK–7 yield in the presence of IONs@APTES and in the absence of nanoparticles. Values are mean ± S.E. of three replicates. An *asterisk* indicates a significantly different value from the control (*p* < 0.05).

The MK–7 specific yield (Figure 7) was significantly higher in the presence of IONs@APTES in the range of 100 to 500 µg/mL as compared to *B. subtilis* cells growing in the absence of nanoparticles (*p* < 0.05). Therefore, decoration of *B. subtilis* cells with IONs@APTES significantly influences MK–7 yield in a concentration-dependent manner. The highest MK–7 specific yield of 0.70 was obtained when *B. subtilis* cells were grown in the presence of 200 µg/mL of IONs@APTES. The MK–7 yield in the absence of nanoparticles was found to be 0.49. Treatment with 200 µg/mL IONs@APTES increased the *B. subtilis* MK–7 fermentation yield by approximately 43%. Therefore, 200 µg/mL of IONs@APTES can be taken as the optimum IONs@APTES concentration to obtain high MK–7 specific yield and high overall productivity during *B. subtilis* fermentation.

### 2.6. Monitoring the Production of MK–7 in the Presence f IONs@APTES

**Figure 8:**
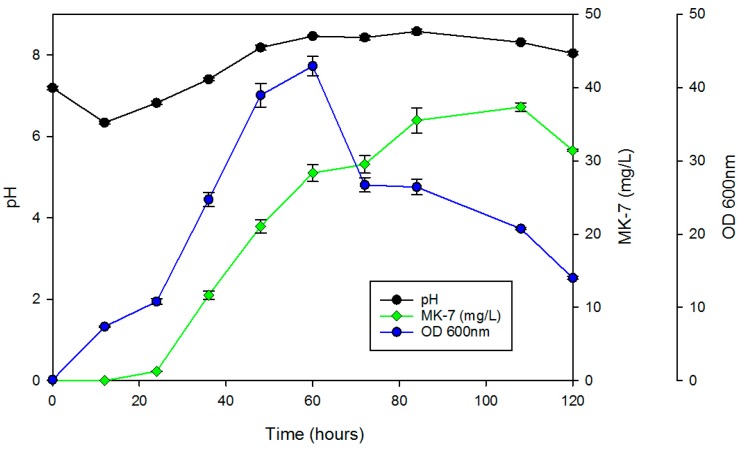
Time course of *B. subtilis* fermentation in the presence of 200 µg/mL IONs@APTES. Error bars indicate standard error of means.

Figure 8 shows the results of a time-course study of MK–7, cell growth and pH of *B. subtilis* (ATCC 6633) in the presence of 200 µg/mL IONs@APTES. Growth increased in a time-dependent manner for about 60 h and reached a maximum cell density of 42.93 (OD_600_). MK–7 biosynthesis was also started after 24 h of fermentation and increased during the logarithmic phase. MK–7 production still increased after *B. subtilis* growth reached its highest level. A maximum MK–7 concentration of 37.36 mg/L was observed when the cell growth was already declining. The pH of the medium decreased from 7.19 to 6.34 during the first 12 h, and increased gradually up to 8.58 after 84 h, following which it again decreased to 8.04 by the end of fermentation.

Treatment with different concentrations of IONs@APTES significantly affected the MK–7 production (*p* < 0.05). In comparison to untreated cells, *B. subtilis* cells decorated with IONs@APTES showed a significant increase in both MK–7 production and yield. It was, however, apparent that the increase in MK–7 concentration is not merely a reflection on cell density. Binding of IONs@APTES to *B. subtilis* might have changed the state or composition of cell membranes, resulting in enhanced secretion of MK–7 to the fermentation medium [7,20].

Most importantly, IONs@APTES did not show any negative effect on *B. subtilis* growth within the tested concentrations, indicating that IONs@APTES are clearly compatible with *B. subtilis* cells. The attachment of NPs to bacteria are governed by physico-chemical interaction between IONs@APTES and *B. subtilis* cells. While some studies indicate a toxicity effect of positively charged NPs on bacteria, other studies report the attachment without showing any detrimental effect on microorganisms [12]. The interaction of IONs@APTES with *B. subtilis* cells therefore provides a brand-new domain for enhancing MK–7 production in *B. subtilis* fermentation without resulting in any harmful effect on bacterial growth. Enhancing the production of MK–7 in *B. subtilis* (ATCC 6633) through the application of nanotechnology has been previously attempted with naked IONs, as well as IONs coated with l-Lysine [7,9], under the same experimental conditions. However, decoration of *B. subtilis* cells with naked IONs and l-Lysine-coated IONs resulted in lower MK–7 concentrations as compared to untreated cells during a 5-day fermentation course [7]. Therefore, in comparison to naked IONs and l-Lysine-coated IONs, IONs@APTES provide a perfect platform for enhancing the MK–7 yield and the productivity of the fermentation system. 

According to monitoring studies using 200 µg/mL IONs@APTES, production of MK–7 started after 24 h, and reached its maximum of 37.36 mg/L after 108 h. The results are in agreement with previous studies [21,22] indicating that the production of MK–7 is partly growth-associated. Time course fermentation studies showed that the pH of the medium decreased from 7.19 to 6.34 during the first 12 h, and increased gradually to 8.18 after 48 h. This increase in pH might be a result of the production of ammonia during the course of fermentation [23]. However, the increase in pH showed no negative impact on the MK–7 yield or bacterial growth. 

## 3. Materials and Methods

### 3.1. Materials

FeCl_3_·6H_2_O, FeSO_4_·4H_2_O. 3–aminopropyltriethoxysilane, ammonium hydroxide, yeast extract, glutaraldehyde 25% aqueous solution, sodium cacodylate Buffer (pH 6.5), peptone from soy meal, ethanol absolute for analysis, 2-propanol and *n*-hexane were purchased from Merck (Roger, AR, USA). Lipase enzyme was obtained from Novozymes (Bagsvaerd, Denmark). Pure MK–7 standard (97.6%) was purchased from ChramoDex (Boulder, CO, USA) for calibration and HPLC analysis. *Bacillus subtilis* (ATCC 6633) was obtained from the New Zealand reference culture collection (Upper Hutt, New Zealand). 

### 3.2. Synthesis of Iron Oxide Nanoparticles Surface Functionalized with APTES

IONs were synthesized by co-precipitation of ferric and ferrous ions with ammonium hydroxide under nitrogen atmosphere as described previously [7,9,12,24,25,26,27,28,29]. Briefly, FeCl_3_·6H_2_O (1.17, 3.8 mmol) and FeSO_4_·7H_2_O (0.74 g, 2.2 mmol) were dissolved in 50 mL of distilled water and the solution was vigorously stirred at 70 °C under nitrogen atmosphere. While stirring was continued, ammonium hydroxide solution (5 mL) was rapidly injected to the mixture. The reaction was continued for 1 h and the resultant black precipitate was separated by centrifugation, washed with boiled distilled water, oven dried at 50 °C overnight, and stored under inert atmosphere. APTES coating was carried out as described previously [12,13]. Briefly, naked ION particles (0.7 g) were dissolved in 25 mL solution of ethanol: water, 1:1 (*v*/*v*) and sonicated for 10 min to get uniform dispersion while were kept in ice bath. APTES solution (2.8 mL) was injected into the mixture under N_2_ atmosphere, while maintaining the temperature of the water bath at 40 °C. The reaction was followed for 2 h at 40 °C with stirring. The resulting particles were finally precipitated by centrifugation and washed with absolute ethanol and deionised water and oven dried at 50 °C overnight.

### 3.3. Characterization of Iron Oxide Nanoparticles Coated with APTES

The size and morphology of nanoparticles were measured by TEM (Philips, CM10: HT 100 Kv, Eindhoven, The Netherlands). The crystal structure of IONs@APTES was determined by XRD (Siemens D5000) with 2-Theta ranging between 20° and 90°. FTIR spectra were obtained using a Bruker, Vertex 70, FTIR spectrometer (Bruker, Kassel, Germany) in the range of 4000–400 cm^−1^.

### 3.4. Growth and MK–7 Production of B. Subtilis Cells in the Presence of IONs@APTES

*B. subtilis* spores grown up to a turbidity of 0.5 McFarland standard (OD_600_ = 0.1) were diluted 1:20 with fresh media and incubated in the presence of varying concentrations of IONs@APTES using the optimum MK–7 fermentation medium developed by Berenjian*,* et al. [30]. McFarland standard was used, as this protocol eliminates the incubation requirement to estimate the number of bacteria. *B. subtilis* cultures without IONs@APTES served as controls. All experiments were conducted with 3 replicates. Vials were incubated for 60 h at 37 °C with shaking at 120 rpm. Optical density measurements were taken at 600 nm.

### 3.5. MK–7 Extraction

MK–7 was extracted from the fermentation medium using a mixture of *n*-hexane and 2-propanol. The volume ratio of medium: 2-propanol: *n*-hexane was 3:2:1. Enzymatic hydrolysis of triglycerides were carried out before extraction by adding 1% lipase powder to the 3 mL of sample and incubating at 37 °C for 45 min in a water bath. A mixture of Ethanol–water (4 mL and 2 mL) was added to the reaction mixture before extracted with 2 mL of 2-propanol and 1mL of *n*-hexane by vigorously vortex-mixing for 1 min and centrifuging for 10 min at 3000 rev/min. 

### 3.6. Menaquinone Analysis by High-Performance Liquid Chromatography (HPLC)

High-performance liquid chromatography (HPLC) HP 2440 (Waters Co., Bedford, MA, USA) with a photo diode array UV detector and Gemini column (5 μm, 250 × 4.6 mm, Phenomenex Co., Torrance, CA, USA) was used for measuring the concentration of MK–7. The mobile phase contained 2-propanol and *n*-hexane (2:1, *v*/*v*) with a flow rate of 0.5 mL/min. The injection loop volume was 20 µL. Detection was carried out with an excitation wavelength of 248 nm. The concentration was calculated by peak area, using a standard curve from serially diluted 0.2 mg/mL standard solution of MK–7 (Chromadex).

### 3.7. Sample Preparation for SEM

Bacterial cultures were grown for 60 h in the presence of varying concentrations of IONs@APTES and in the absence of nanoparticles. Samples were prepared as described previously [12]. Briefly, air-dried bacterial smear was heat-fixed by passing through the flame of a Bunsen burner, and fixed with 2.5% glutaraldehyde in 0.1 M sodium cacodylate buffer with four changes over 30 min in room temperature. Slides were rinsed with normal saline four times over 30 min and the cells were dehydrated through a series of alcohol concentrations (50%, 75%, 95%) for 1 h in each solution and four changes in absolute ethanol for 20 min. Specimens were subjected to critical point drying (Poloron). Samples were then mounted on aluminium stubs and coated with platinum before examining with a Scanning Electron Microscope (Hitachi S-4700, Tokyo, Japan).

### 3.8. Statistical Analysis

Statistical significance was determined by analysis of variance (ANOVA) and Dunnett multiple comparison tests using IBM SPSS statistics 24. Data were reported as the mean ± standard error of three replicates. Mean values were considered significantly different at *p* < 0.05. 

## 4. Conclusions

Cultivation of *B. subtilis* in the presence of IONs@APTES significantly enhanced the MK–7 yield without showing any inhibitory effect on *B. subtilis* growth. This study provides a great promise for synthesis and application of IONs@APTES in cell immobilization in future bioprocess engineering to overcome the low product yield of MK–7. Therefore, it is of utmost importance to consider the results of the present study for further development of an industrial level production system for MK–7 using IONs@APTES.
